# Uptake and tolerability of repeated mucosal specimen collection in two Phase 1 AIDS preventive vaccine trials in Kenya

**DOI:** 10.1186/1742-4690-9-S2-P122

**Published:** 2012-09-13

**Authors:** G Mutua, G Omosa-Manyonyi, H Park, P Bergin, D Laufer, PN Amornkul, J Lehrman, P Fast, J Gilmour, O Anzala, B Farah

**Affiliations:** 1 Kenya AIDS Vaccine Initiative, Nairobi, Kenya; 2 International AIDS Vaccine Initiative, New York, NY, USA; 3 International AIDS Vaccine Initiative Human Immunology Laboratory, London, UK

## Background

Mucosal specimens are useful to evaluate local immune responses in AIDS preventive vaccine trials, but the acceptability and tolerability of mucosal sampling in Africa remains unknown.

## Methods

The Kenya AIDS Vaccine Initiative (KAVI) initiated two AIDS preventive vaccine trials in Nairobi in 2011. After informed consent for a mucosal substudy, participants were asked to provide any of several types of mucosal secretions: saliva, oral fluids, semen, cervico-vaginal and rectal. Specimens were collected at baseline, one month after final vaccination, and at the next scheduled trial visit. A tolerability questionnaire was administered at the final visit.

## Results

Of 80 trial participants, 65(81.3%) consented to the mucosal sub-study and provided at least one specimen, 7/65(10.8%) gave all specimens at least once and 2/65(3.1%) gave all possible specimens at all visits. Saliva and oral fluids were given at all time-points by 62/65(95.4%) participants. Of 48 men, 21(43.8%) provided semen at baseline, 18/21 completed all 3 time-points. Of 17 women, 15(88.2%) gave vaginal sponge and SoftCup specimens at least once; 8/15(53.3%) gave both at all eligible time-points. Rectal sampling was the least acceptable method: 13/65(20%) participants agreed at baseline [4/17 women (23.5%), 9/48 men (18.8%)]. Of these, 4 men and 2 women gave samples at all time-points. The most common reason for accepting mucosal sampling was a desire to contribute to HIV research and for refusal, embarrassment/emotional discomfort.

## Conclusion

Repeated saliva, oral fluid, semen and cervico-vaginal mucosal sampling in AIDS vaccine preventive trials in Kenya is feasible; this study however re-affirms the challenge of repeated rectal mucosal sampling in low-risk participants, noted in an earlier observational study at KAVI (AIDS Vaccine 2010 P10.07). Possible explanations include cultural and religious reasons contributing to embarrassment and emotional discomfort in low-risk participants. Including more qualitative research in vaccine trials with mucosal sampling could help elucidate these findings.

